# Vitamin D deficiency associated with Crohn’s disease and ulcerative colitis: a meta-analysis of 55 observational studies

**DOI:** 10.1186/s12967-019-2070-5

**Published:** 2019-09-23

**Authors:** Xi-Xi Li, Yang Liu, Jie Luo, Zhen-Dong Huang, Chao Zhang, Yan Fu

**Affiliations:** 1 Center for Evidence-Based Medicine and Clinical Research, Taihe Hospital, Hubei University of Medicine, No. 32, South Renmin Road, Shiyan, 442000 China; 2 Zhejiang Chinese Medical University, No. 548, Binwen Road, Zhengjiang, 310053 China; 3 Department of General Surgery, Taihe Hospital, Hubei University of Medicine, No. 32, South Renmin Road, Shiyan, 442000 China

**Keywords:** Inflammatory bowel disease, Crohn’s disease, Ulcerative colitis, Vitamin D deficiency, Meta-analysis

## Abstract

**Purpose:**

To investigate the association of serum levels of 25(OH)D and 1,25(OH)_2_D_3_ in healthy and non-healthy controls with Crohn’s disease (CD) and ulcerative colitis (UC).

**Methods:**

Three electronic databases: PubMed, EMbase and EBSCO*host* CINAHL, were searched for observational studies to measure the relationship between serum levels of vitamin D (VitD) and CD (or UC).

**Results:**

Fifty-five studies were included in the meta-analysis. We found that mean serum 25(OH)D levels in patients with CD were significantly lower than those in healthy controls (MD: − 3.17 ng/mL; 95% CI − 4.42 to − 1.93). Results from the meta-analysis examining 1,25(OH)_2_D_3_ levels in Crohn’s patients revealed higher levels in the CD group than in healthy (MD: 3.47 pg/mL; 95% CI − 7.72 to 14.66) and UC group (MD: 5.05 pg/mL; 95% CI − 2.42 to 12.52). Serum 25(OH)D levels were lower in the UC group than in the healthy control group (MD: − 2.52 ng/mL; 95% CI − 4.02 to − 1.02). In studies investigating the level of 1,25(OH)_2_D_3_ in UC and healthy control groups, the level of 1,25(OH)_2_D_3_ in the UC groups were found to be higher than that in the control groups (MD: 3.76 pg/mL; 95% CI − 8.36 to 15.57). However, the 1,25(OH)_2_D_3_ level in patients with UC was lower than that in CD groups (MD: − 6.71 pg/mL; 95% CI − 15.30 to 1.88). No significant difference was noted between CD patients and UC patients in terms of average serum 25(OH)D levels.

**Conclusions:**

This study found that VitD levels were inversely related to CD and UC. Serum levels of 25(OH)D were lower in patients with CD and UC than in healthy people, and more than half of the patients had insufficient vitamin D levels. The serum level of 1,25(OH)_2_D_3_ in both the CD and UC groups was higher than that in healthy people.

## Introduction

Inflammatory bowel disease (IBD), including the two major forms: Crohn’s disease (CD) and ulcerative colitis (UC), is a chronic, relapsing–remitting systemic disease that typically begins in young adulthood and lasts throughout life. Although progress has been made in understanding these diseases, their etiology is unknown [1]. CD is a chronic inflammatory disease characterized by discontinuously affected areas with transmural, granulomatous inflammation and/or fistula, and can affect any region in the digestive tract, from the mouth to the anus, but is more likely to involve the small and large intestines (especially the ileocecum) and the perianal region. UC is a diffuse, non-specific inflammatory disease of unknown cause that continuously affects the proximal colonic mucosa from the rectum and often forms erosions and/or ulcers [2]. Since there is currently no cure for IBD, medical therapy remains the primary treatment for achieving and maintaining remission [3].

Currently, there is general agreement that variations in a patient’s genetic make-up, broad changes in the surrounding environment, alterations in the composition of gut microbiota, and the reactivity of the intestinal mucosal immune response are at the foundation of IBD pathogenesis [4]. Vitamin D (VitD) is known to induce and maintain the alleviation of IBD through anti-bacterial and anti-inflammatory actions and repair of the intestinal mucosal barrier [5, 6]. VitD belongs to a family of fat-soluble secosteroid hormones and comprises two major forms: VitD_2_ (ergocalciferol) and VitD_3_ (cholecalciferol) [7]. VitD_3_ is hydroxylated in the liver into 25(OH)D and subsequently in the kidney into 1,25(OH)_2_D_3_ [8]. VitD has been shown to target the three major components of the gastrointestinal epithelial barrier, intestinal immunity and intestinal microflora and has multiple effects on intestinal health [9]. Through active intestinal signaling, which has immunomodulatory and immunosuppressive effects on inflammatory and inhibitory markers of IBD, VitD interferes with the immune response to bacterial activity, antigen presentation and adaptive and innate immune regulation. Therefore, VitD may affect the incidence and progression of UC and CD [10–12]. While attempting to rule out VitD deficiency in patients with IBD due to reduced physical activity, sunlight exposure, malnutrition, inadequate dietary intake of VitD, or lower bioavailability, some studies [3, 13, 14] have found that VitD deficiency is also common in newly diagnosed IBD patients. Thus, VitD deficiency may play a role in the development of IBD and its severity. Other studies, however, have taken the opposite view of the relationship [15] between VitD and IBD and have left the controversy unresolved for patients with CD [16] and UC [17, 18]. Therefore, to explore this controversy we performed a pooled meta-analysis to investigate and determine the status of VitD in the serum of healthy and non-healthy controls and to study the association between serum 25(OH)D and 1,25(OH)_2_D_3_ concentrations and an IBD diagnosis (both UC and CD).

## Materials and methods

### Search strategy

All studies were obtained by searching PubMed, EMbase and EBSCO*host* CINAHL for articles that were published through April 8, 2019. Detailed search strategies are shown in Additional file 1: Method S1.

### Inclusion and exclusion criteria

Studies were eligible for analysis if they met the following criteria: (1) all included studies were limited to observational investigations in English; (2) serum VitD levels were detected in CD or UC patients; (3) when several trials from the same authors were identified as duplicates, we only included the most recent trial with the largest number of patients or with a longer follow-up period. The healthy control group was defined as those without CD or UC, and the non-healthy control was defined as patients diagnosed with CD or UC, but it was different from the exposed group.

Exclusion criteria included: (1) studies conducted exclusively on patients with IBD diseases, but not CD or UC; (2) studies that did not present any distinct serum levels of VitD; (3) studies that did not include the standard deviation of mean serum levels of VitD, and attempts to get these values by contacting the authors through email were unsuccessful; (4) non-full-text English articles.

### Data extraction

For each included study, two investigators independently extracted the following essential information: name of the first author, publication year, study design, disease type, country, age, sex, use of any matching or adjustment approach, maturity, VitD assessment tool, VitD deficiency definition, and VitD supplementation. Disagreements were resolved through discussion or from a third party.

### Study quality assessment

The quality of each study from case–control and cohort study in the meta-analysis was assessed using the Newcastle–Ottawa Scale [19, 20], which ranges from 1 to 9 stars and judges each study according to three aspects: selection of the study groups; the comparability of the groups; and, the ascertainment of the outcome of interest. For the cross-sectional study, the quality assessment method from were employed by The Joanna Briggs Institute Critical Appraisal tools for use in JBI Systematic Reviews [21].

### Data analysis

For continuous data, the mean difference (MD) and 95% confidence interval (CI) were calculated [22]. If different measurement indices adopted different tools in the various studies, the standardized mean difference (SMD) was used [22]. A fixed-effects model was used when there was no significant heterogeneity (P > 0.1, I^2^ < 40%), otherwise, a random-effect model was employed [23]. To further explore sources of heterogeneity, subgroup analyses were performed according to age, VitD measurement tools, VitD supplementation, and study design based on both healthy and non-healthy populations using 25(OH)D and 1,25(OH)_2_D_3_. Publication bias was assessed by visual inspection of funnel plots [24]. Sensitivity analysis was used to explore the extent to which extrapolation might depend on a particular study or group of studies, excluding small sample studies (both groups < 30) and studies with low study scores (< 5) to discuss the sources of heterogeneity. R 3.4.4 software was performed for all statistical analyses.

## Results

### Study characteristics

**Fig. 1:**
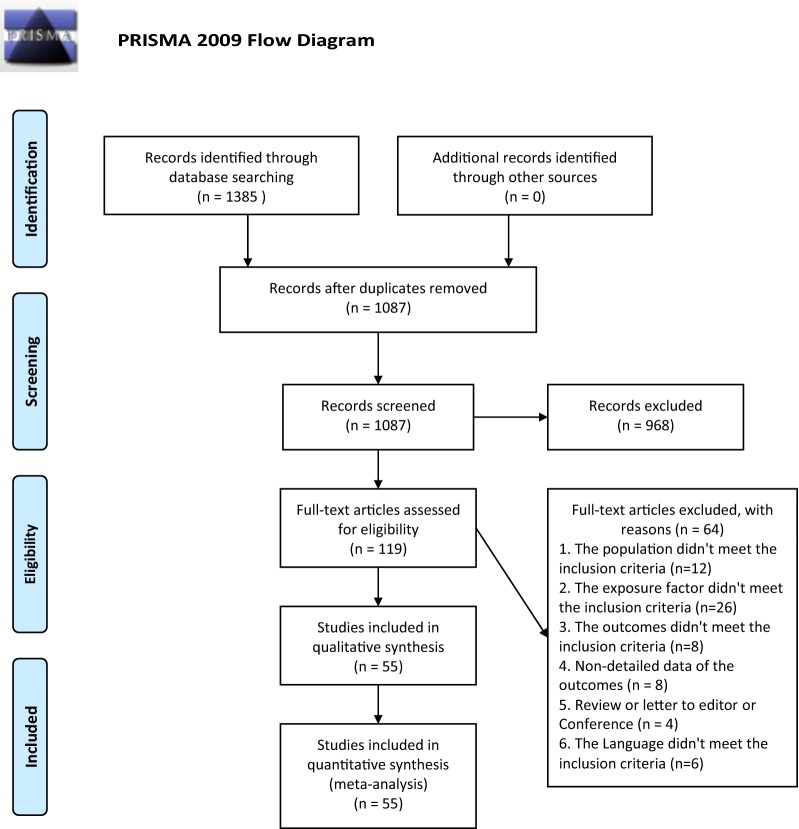
Meta-analyses (PRISMA) flow diagram depicting the process of identification and inclusion of selected studies

The literature search identified 1385 individual studies. After removing 298 duplicates, 1087 potentially relevant studies were selected on the basis of the abstract, and of these, 119 full texts were assessed for eligibility. In total, 55 publications [16, 18, 25–77] were included in the meta-analysis (Fig. 1).

A total of 19 cohort studies [18, 34, 38, 41, 50–56, 64, 67, 68, 71, 73, 74, 76, 77], 22 case–control studies [16, 25–29, 31–33, 35, 42, 43, 46, 49, 59–63, 66, 69, 70] and 14 cross-sectional studies [30, 36, 37, 39, 40, 44, 45, 47, 48, 57, 58, 65, 72, 75] were included in the analysis. The total number of participants was 5123 patients and 3033 healthy controls. Different studies investigated a range of VitD deficiency values: some used 20 ng/mL [16, 18, 35, 36, 40, 42, 48, 51, 54, 55, 64, 65, 67, 68, 72–75] (50 nmol/L) (n = 18); Other studies used 15 ng/mL [31, 37, 46, 49, 57] (n = 5), 10 ng/mL [32, 41, 50, 62] (n = 4), 12 ng/mL [59–61] (n = 3) or 30 ng/mL [56, 65] (n = 2). The mean difference in 25(OH)D concentrations among patients with CD compared with healthy controls ranged between − 16.58 and 8.19 ng/mL and between − 8.98 and 7.50 ng/mL for non-healthy controls. The values for 1,25(OH)_2_D ranged between − 11.50 and 34.79 pg/mL for healthy controls and between − 5.70 and 22.80 pg/mL for non-healthy controls. The mean difference between 25(OH)D levels among patients with UC compared with healthy controls ranged between − 18.07 and 2.90 ng/mL and between − 4.25 and 8.98 ng/mL for non-healthy controls. The values for 1,25(OH)_2_ D_3_ ranged between − 8.24 and 25.25 pg/mL for healthy controls and between − 22.80 and 5.70 pg/mL for non-healthy controls. Most of the studies matched cases and controls for age and gender. A few studies used race, body mass index, weight and smoking as additional matching variables and most did not include VitD supplements.

Table 1 shows that the quality scores of the included studies ranged from 2 to 7, with a median of 5. Thirty-two studies [16, 18, 29–31, 33–35, 37–40, 44, 45, 47–49, 51, 52, 54, 55, 61, 62, 64–66, 70, 73–77] were considered high quality and the others [25–28, 31, 36, 41–43, 46, 50, 53, 56–60, 63, 67–69, 71, 72] were low quality.

**Table 1:** Characteristics of studies included in the meta-analysis

Study	Year	Study design	Country	Disease	Totle, CD/UC/control	Female, CD/UC/control	Matching or adjustment	Maturity (CD/UC/control)	Vitamin D assessment tool	Vitamin D deficiency definition (ng/mL for 25(OH)D, pg/mL 1,25(OH)2D)	Vitamin D supplementation	Quality score
Driscoll [25]	1982	Case–control	US	CD	82/–/40	NR/–/NR	NR	> 18	CPBA	Normal: 15.1–27.9	Yes	5
Harries [26]	1985	Case–control	Wales	CD and UC	40/20/9	21/9/6	NR	38.75 ± 15.42/45 ± 17/–	RIA	NR	No	5
Westarp [69]	1987	Case–control	Canada	CD	39/–/64	25/–/37	NR	9.3 ± 0.3	CPBA	NR	No	5
Martin [70]	1994	Case–control	Italy	CD	20/–/12	0/–/0	Age	38.8 ± 9.94/–/43 ± 14	HPLC	NR	No	6
Pollak [27]	1998	Case–control	Israel	CD and UC	63/41/–	23/21/–	Age, sex	37.7 ± 14.5 (IBD)/34.6 ± 11.2	RIA	Normal: 10–45	No	4
Gokhale [28]	1998	Case–control	US	CD and UC	58/37/–	22/17/–	NR	14.3 ± 2.9/13.7 ± 3.5/–	CPBA	25(OH)DNormal: 10–60; 1,25(OH)2DNormal (2–12 years): 10.8–90.2	No	5
Ardizzone [29]	2000	Case–control	Italy	CD and UC	51/40/30	30/15/16	Age, sex	38.7 ± 13.2/34.4 ± 12.5/39.4 ± 11.6	RIA	25(OH)DNormal: 15–40; 1,25(OH)2DNormal: 14–50	No	7
Jahnsen [30]	2002	Cross-sectional	Norway	CD and UC	60/60/–	36/36/–	Age, sex	36 ± 16.5/38 ± 13.5/–	HPLC + RIA	25(OH)DNormal: 12–44; 1,25(OH)2DNormal: 19–56	No	7
Haderslev [31]	2003	Case–control	Denmark	CD and UC	42/–/384	24/–/NR	NR	50.3 ± 12.3	RIA	Deficiency: < 15	No	4
Tajika [32]	2004	Case–control	Japan	CD and UC	33/11/15	8/5/7	Age, sex	37.6 ± 7.5/47.6 ± 12.4/37.7 ± 10.0	CPBA + RIA	25(OH)DNormal: 10–55; deficiency: ≤ 10; 1,25(OH)2DNormal: 20–60	No	6
Duggan [33]	2004	Case–control	Ireland	CD	44/–/44	29/–/29	NR	36.9 ± 11.1/–/36.7 ± 11.0	ELISA	NR	6.7 ± 5.1/6.7 ± 4.8 μg	6
Abreu [34]	2004	Cohort	US	CD and UC	138/29/96	63/12/NR	NR	37.7 ± 1.1/38.1 ± 3.3/40.0 ± 1.0	CPBA	Elevated 1,25(OH)2D: > 60; normal 1,25(OH)2D: < 60	No	6
McCarthy [35]	2005	Case–control	Ireland	CD	44/–/44	29/–/29	Age, sex	36.9 ± 11.1/–/36.7 ± 11.1	ELISA	Insufficiency: < 32; sufficiency: > 32; replete: > 20; mild deficiency: 10–20; moderate deficiency: 5–10; severe deficiency: < 5	2.5–20 μg/day	6
Gilman [36]	2006	Cross-sectional	Ireland	CD and UC	47/26/73	NR/NR/NR	Age, sex	> 18	ELISA	Deficiency: < 20	No	5
Pappa [37]	2006	Cross-sectional	US	CD and UC	94/36/–	43/20/–	NR	15 ± 3/14 ± 4/–	NR	Deficiency: ≤ 15; severe deficiency: ≤ 8	Yes	3
Sinnott [38]	2006	Cohort	US	CD and UC	30/18/–	14/9/–	Age, sex	48.0 ± 12.0/48.9 ± 15.7/–	NR	NR	No	4
Vagianos [39]	2007	Cross-sectional	canada	CD and UC	84/42/–	52/25/–	NR	37.6 ± 14.3/36.6 ± 12.9/–	CPBA	Normal: 14–80; deficiency: 20–30	Yes	4
Kuwabara [40]	2008	Cross-sectional	Japan	CD and UC	29/41/–	9/17/–	NR	32.2 ± 6.7/39.3 ± 14.6/–	RIA	Deficiency: < 20; insufficiency: 21–29	No	3
Leslie [41]	2008	Cohort	Canada	CD and UC	56/45/–	NR/NR/–	NR	> 18	RIA	Optimal: > 30; marginally deficient: 20–30; insufficiency: 10–19; deficiency: < 10	No	6
Souza [71]	2008	Cohort	Brazil	CD and UC	39/37/40	18/25/24	NR	32.1 ± 8.7/35.0 ± 8.5/34.0 ± 7.0	RIA		No	6
Joseph [42]	2009	Case–control	India	CD and UC	34/34/–	10/10/–	Age, sex	39.2 ± 12.9/38.9 ± 13.4 (IBS)	RIA	Deficiency: < 20; insufficiency: 20–32; adequate: > 32	No	6
Kumari [43]	2010	Prospective case–control	Georgia	CD	4/–/4	0/–/0	Age	35.5 ± 9.75/–/42.40 ± 5.13	ELISA	Insufficiency: < 30	240.50 ± 119.92/211.60 ± 132.11 (IU)	6
EI-Matary [44]	2011	Cross-sectional	Canada	CD and UC	39/21/56	20/11/31	Age, sex, ethnicity	12.2 ± 3.2/12.4 ± 3.7/11.3 ± 4.2	CPBA	Optimum: ≥ 32	No	3
Levin [45]	2011	Cross-sectional	Australia	CD and UC	70/8/–	NR/NR/–	NR	12.6 ± 3.5	CLIA	NR	No	3
Pappa [47]	2011	Cross-sectional	US	CD and UC	288/143/–	127/78/–	Age, sex, ethnicity	15.9 ± 3.1/15.4 ± 3.3/–	CLIA	Optimum: ≥ 32	Yes	4
Atia [48]	2011	Cross-sectional	US	CD and UC	43/80/–	3/7/–	NR	61.4 ± 14.7/66.5 ± 11.5/–	CLIA	Deficiency: < 20; insufficiency: < 30	No	2
EI-Hodhod [46]	2012	Case–control	Egypt	CD and UC	20/27/50	2/13/9	Age, sex	10.49 ± 3.34/12.77 ± 1.71/12.8 ± 3.77	RIA	Deficiency: < 15; severe deficiency: < 8	No	6
Suibhne [49]	2012	Case–control	Ireland	CD	81/–/70	48/–/42	Age, sex,socio-economic status.	36.43 ± 11.00/–/36.34 ± 9.53	RIA	2cut-points: (1) deficiency: < 20; (2) deficiency: < 32	200–400 IU; ≥ 800 IU	5
Hassan [50]	2012	Cohort	Iran	CD and UC	26/34/–	7/10/–	NR	34 ± 18/30 ± 11/–	RIA	Sufficiency: ≥ 30; insufficiency: 11–29; deficiency: ≤ 10 ng/mL	No	7
Chatu [51]	2012	Retrospective cohort	UK	CD and UC	107/61/–	NR/NR/–	NR	34.98 ± 14.36(IBD)/–	CPBA	Normal: ≥ 20; deficiency: < 20; severe: < 10	No	4
Fu [52]	2012	Cohort	Canada	CD and UC	40/60/–	18/32/–	NR	40 ± 13.2/42.1 ± 13.9/–	RIA	Hypovitaminosis: < 20	No	5
Salacinski [53]	2012	Cohort	US	CD	19/–/19	10/–/10	Age, sex	44.16 ± 10.28/–/41.68 ± 11.19	HPLC	Low 25(OH)D levels: < 20 ng/mL; insufficient: 20–32 ng/mL	No	3
Garg [54]	2013	Cohort	Australia	CD and UC	40/31/23	18/14/13	Sunlight exposure	41 ± 13.25/44 ± 15/42 ± 11.5	CLIA	Sufficiency: ≥ 30; insufficiency: 20–30; deficiency: < 20	795/927/473(UI)	6
Prosnitz [55]	2013	Cohort	US	CD	78/–/221	34/–/109	Anthropometry, body composition, pubertal development weight and height	12.7 ± 2.8/–/13.5 ± 4.4	RIA	Deficiency: < 20	No	7
Miznerova [56]	2013	Cohort	Slovakia	CD and UC	46/30/–	25/15/–	NR	36 ± 12.75/47 ± 13.5/–	ECLIA	Deficiency: < 30; very low: < 10	No	4
Grunbaum [17]	2013	Case–control	Canada	CD and UC	34/21/48	21/13/38	Age, sex, ethnicity, weight	39.9 ± 12.3/44.2 ± 13.7/39.6 ± 13.8	RIA	Replete: ≥ 30; insufficiency: 20–29; deficiency: < 20; severely deficiency: < 10	932.4/1020.8 (IU)	6
Jorgensen [72]	2013	Cross-sectional	Denmark	CD	182/–/62	57/–/52	NR	36 ± 10.2/–/32 ± 11	LC–MS	Deficiency: < 20	Yes	5
Middleton [57]	2013	Cross-sectional	US	CD	52/–/40	20/–/25	NR	17.0 ± 0.9/–/11.0 ± 2.5	CLIA + LC–MS	Deficiency: ≤ 15; insufficiency: < 32	No	5
Lorinczy [58]	2013	Cross-sectional	Hungary	CD and UC	128/41/–	NR/NR/–	Age, sex	35.8 ± 12.0	CLIA	NR	No	5
Alkhouri [59]	2013	Case–control	US	CD and UC	46/12/61	14/6/31	Age, sex	12.1 ± 4.1/12.3 ± 3.5/12.1 ± 3.6	NR	Deficiency: < 12; severely deficiency: < 4	No	4
Bruyn [60]	2014	Prospective case–control	Netherlands	CD	98/–/43	68/–/NR	NR	36 ± 10.2/–/32 ± 7.3	CLIA	Normal: ≥ 30; insufficiency: 20–30; deficiency: < 20	Yes	5
Dumitrescu [61]	2014	Prospective case–control	Romania	CD and UC	14/33/94	6/16/44	Age, sex	36 ± 9/42 ± 14/42 ± 12	HPLC	Sufficiency: ≥ 30; insufficiency: 20–30; deficiency: < 20	No	7
Tan [62]	2014	Case–control	China	CD and UC	107/124/122	61/39/55	Age, sex	38.0 ± 15.3/39.6 ± 14.4/39.43 ± 12.71	ELISA	Sufficiency: ≥ 20; insufficiency: 10–20; deficiency: < 10	No	7
Oikonomou [63]	2014	Case–control	Greece	CD	44/–/20	22/–/14	NR	31 ± 8/–/30 ± 6.75	CLIA	NR	No	4
Veit [64]	2014	Cohort	US	CD and UC	40/18/116	16/11/67	Age	16.61 ± 2.20/16.13 ± 1.99/14.56 ± 4.35	CPBA	Sufficiency: ≥ 30 ng/mL; insufficiency: 20–29.9; deficiency: < 20 ng/mL	No	7
Basson [65]	2015	Cross-sectional	South Africa	CD	186/–/199	NR/–/NR	NR	47.35 ± 14.20/–/34.11 ± 15.16	CLIA	Deficiency: ≤ 20 or 29 ng/mL	No	7
Thorsen [66]	2016	Case–control	Danish	CD and UC	155/210/384	69/114/196	NR	13.65 ± 2.24/14.30 ± 4.48/NS	LC–MS	NR	No	7
Schäffler [67]	2017	Cohort	Germany	CD and UC	123/85/–	NR/NR/–	NR	NR	NR	Deficiency: < 50 nmol/mL; insufficiency: < 75 nmol; normal: ≥ 75 nmol	No	4
Opstelten [68]	2018	Multicenter cohort	UK	CD and UC	72/169/144 338	56/82/112 164	Age, sex	49.55 ± 4.62/51.63 ± 2.20/48.94 ± 3.37; 51.61 ± 1.96	LCMS	Deficiency: ≤ 50 nmol/mL; insufficiency: 50–75 nmol/mL; sufficiency: ≥ 75 nmol/mL	No	5
Scotti [73]	2018	Cohort	Italy	CD and UC	126/174/–	56/76/–	Age, sex	51 ± 16.7/51 ± 17.9/–	ELISA	Severe deficiency: ≤ 10 ng/mL; deficiency: 11–20 ng/mL; insufficient levels 21–30 ng/mL; adequate levels > 30 ng/mL	No	6
Garg [74]	2018	Cohort	Australia	UC	–/17/8	–/7/3	Age, sex	–/47.26 ± 11.55/50.75 ± 8.95	LCMS	Deficiency: < 50 nmol/mL	40000 IU/week	7
Caviezel [75]	2018	Cross-sectional	Switzerland	CD and UC	99/57/–	48/31/–	Age, sex	41.2 ± 14.5/41.5 ± 13.6/–	CPBA	Deficiency: < 50 nmol/mL	NO	7
Kyoung [18]	2018	Retrospective cohort	Korea	CD and UC	42/45/–	17/13/–	Age, sex	40.9 ± 15.6/48.5 ± 13.7/–	CLIA	Deficiency: < 20 ng/mL	No	6
Strisciuglio [76]	2018	Cohort	Italy	CD and UC	12/21/18	17/8	Age, sex	11 ± 3.25 (IBD)/9.2 ± 2.5	ELISA	NR	No	7
Grag [77]	2019	Cohort	Australia	CD and UC	20/15/14	8/5/7	Age, sex	43.75 ± 11.75/42.75 ± 11.75/48.25 ± 13.56	NR	NR	Yes	8

*CPBA* competitive protein binding assay, *RIA* radioimmunoassay, *ECLIA* electrochemiluminescence immunoassay, *ELISA* enzyme-linked immunosorbent assay, *CLIA* chemiluminescence, *HPLC* high performance liquid chromatography, *LC–MS* liquid chromatograph mass spectrometer, *NR* not reported

### Findings of the meta-analysis for serum 25(OH)D levels in Crohn’s patients

**Fig. 2:**
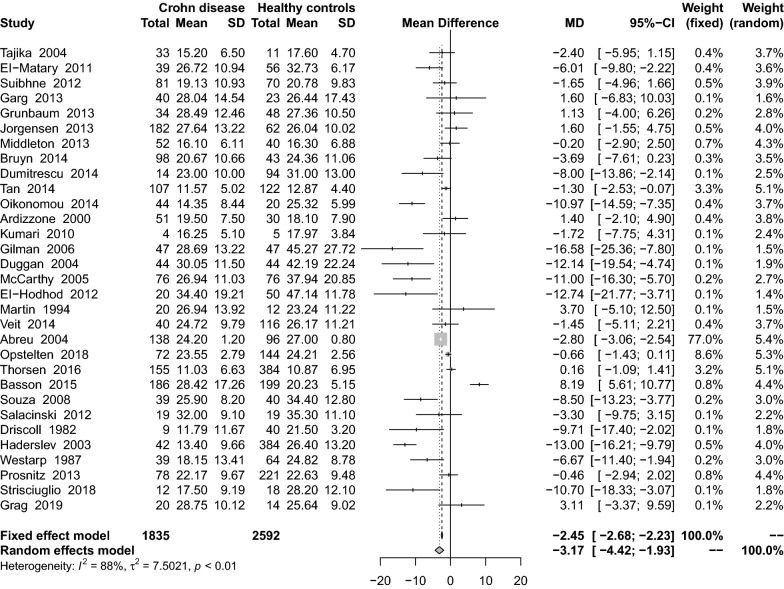
Mean difference of serum 25(OH)D levels among patients with Crohn’s disease compared with healthy controls

A total of 31 studies [16, 25, 29, 31–36, 43, 44, 46, 49, 53–55, 57, 60–66, 68–72, 76, 77] were conducted on serum 25(OH)D levels in CD and healthy controls, and we conducted a meta-analysis of 29 effect values. We found mean serum 25(OH)D levels in patients with CD were significantly lower than in healthy controls (MD: − 3.17 ng/mL; 95% CI − 4.42 to − 1.93) (Fig. 2). There was significant heterogeneity among the studies (I^2^ = 88%, P < 0.01). Subgroup analysis (Table 2) showed that the mean serum 25(HO)D levels in adult CD patients was statistically significant compared to the control group (MD: − 3.22 ng/mL; 95% CI − 4.75 to − 1.70) and children (MD: − 3.16 ng/mL; 95% CI − 5.54 to − 0.77). Compared with the control group, CLIA (MD: − 1.32 ng/mL; 95% CI − 8.89 to 6.26), ELISA (MD: − 8.29 ng/mL; 95% CI − 13.83 to − 2.76) and RIA (MD: − 3.22 ng/mL; 95% CI − 4.46 to − 0.13) were statistically significant, while CPBA, HPLC and LC–MS showed no statistical significance. Both the presence and absence of VitD supplementation was statistically significant (MD: − 1.49 ng/mL; 95% CI − 4.40 to 1.42) and (MD: − 3.46 ng/mL; 95% CI − 4.90 to − 2.03), respectively. In regards to study design, case–control studies (MD: − 4.95 ng/mL; 95% CI − 7.18 to − 2.72) and cohort studies (MD: − 2.11 ng/mL; 95% CI − 3.69 to − 0.53) reported statistically significant results to the control group, but the cross-sectional studies did not find statistically significant differences. In sensitivity results, the residual results were unchanged after excluding small sample studies (MD: − 3.48 ng/mL; 95% CI − 4.78 to − 2.17) or excluding studies with lower quality score (MD: − 2.12 ng/mL; 95% CI − 3.34 to − 0.90).

**Table 2:** Results of subgroup analysis

Subgroup analyses	Crohn disease				Ulcerative colitis			
	No. of effect sizes	Mean (95% CI)	P for mean	I^2^ (%)	No. of effect sizes	Mean (95% CI)	P for mean	I^2^ (%)
25(OH)D among disease patients and healthy controls								
Maturity								
Adults (> 18 years old)	24	− 3.22 (− 4.75 to − 1.70)	< 0.01	90	11	− 2.38 (− 4.20 to − 0.56)	< 0.01	85
Children (< 18 years old)	8	− 3.61 (− 4.89 to − 2.32)	< 0.01	90	4	− 4.45 (− 9.42 to 0.53)	< 0.01	78
Vitamin D assessment tool								
CLIA	5	− 1.32(− 8.89 to 6.26)	< 0.01	95	2	− 3.10 (− 7.50 to 1.30)	0.2	38
CLIA + LC–MS	1	− 0.20 (− 2.90 to 2.50)	NR	NR	0	NR	NR	NR
CPBA	5	− 4.28 (− 6.40 to − 2.16)	0.06	55	1	− 1.10 (− 2.31 to 0.11)	NR	NR
ELISA	6	− 8.29 (− 13.83 to − 2.76)	< 0.01	85	3	− 8.22 (− 16.62 to 0.19)	< 0.01	86
HPLC	3	− 3.23 (− 9.40 to 2.95)	0.09	58	1	− 7.00 (− 11.58 to − 2.42)	NR	NR
LC–MS	3	− 0.35 (− 0.99 to 0.29)	0.25	27	2	− 0.15 (− 0.57 to 0.27)	0.77	0
RIA	8	− 4.46 (− 9.05 to 0.13)	< 0.01	90	4	− 4.52 (− 12.89 to 3.85)	< 0.01	89
NR	1	3.11 (− 3.37 to 9.59)	NR	NR				
Vitamin D supplementation								
No	24	− 3.46 (− 4.90 to − 2.03)	< 0.01	91	12	− 3.29 (− 4.99 to − 1.60)	< 0.01	87
Yes	7	− 1.49 (− 4.40 to 1.42)	< 0.01	66	3	0.72 (− 1.98 to 3.41)	0.95	0
NR	1	− 12.14 (− 19.54 to − 4.74)	NR	NR	0	NR	NR	NR
Study design								
Case–control study	19	− 4.95 (− 7.85 to − 3.11)	< 0.01	89	7	− 2.24 (− 4.59 to 0.11)	< 0.01	79
Cohort study	9	− 2.11 (− 3.69 to -0.53)	< 0.01	82	4	− 2.58 (− 5.29 to 0.13)	< 0.01	89
Cross-sectional study	4	− 0.44 (− 6.76 to 5.87)	< 0.01	93	1	− 18.07 (− 26.50 to -9.64)	NR	NR
25(OH)D among disease patients and non-healthy controls								
Maturity								
Adults (> 18 years old)	28	− 0.84 (− 2.12 to 0.44)	< 0.01	85	26	0.65 (− 0.65 to 1.95)	< 0.01	86
Children (< 18 years old)	9	0.53 (− 2.16 to 3.22)	< 0.01	78	8	0.92 (− 2.05 to 3.90)	< 0.01	79
NR	1	− 1.88 (− 5.52 to 1.76)	NR	NR	1	1.88 (− 1.76 to 5.52)	NR	NR
Vitamin D assessment tool								
CLIA	7	1.66 (− 1.36 to 4.68)	< 0.01	73	6	− 0.81 (− 3.96 to 2.43)	< 0.01	73
CPBA	7	− 0.80 (− 2.79 to 1.20)	< 0.01	76	6	1.94(− 0.03 to 3.91)	< 0.01	78
ECLIA	2	1.34 (0.17 to 2.52)	0.62	0	2	− 1.34 (− 2.52 to − 0.17)	0.23	31
ELISA	4	1.60 (− 5.26 to 2.07)	< 0.01	84	1	0.18 (− 3.65 to 4.01)	NR	NR
HPLC	2	− 3.27 (− 6.35 to 0.19)	0.53	0	1	3.69 (0.34 to 7.04)	NR	NR
LC–MS	2	0.96 (− 0.84 to 2.76)	0.02	80	2	− 0.96 (− 2.76 to 0.84)	0.02	80
RIA	10	− 1.65 (− 5.16 to 1.86)	< 0.01	85	9	1.18 (− 2.61 to 4.98)	< 0.01	87
NR	4	− 2.35 (− 4.91 to − 0.20)	0.67	0	2	2.35 (− 0.20 to 4.91)	0.45	0
Vitamin D supplementation								
No	34	− 0.48 (− 1.70 to 0.74)	< 0.01	84	31	− 0.71 (− 0.63 to -2.05)	< 0.01	85
Yes	4	− 2.36 (− 3.25 to − 1.46)	0.45	0	3	2.36 (1.46 to 3.25)	0.45	19
Study design								
Case–control study	12	− 0.07 (− 1.77 to 1.64)	< 0.01	58	9	0.91 (− 1.09 to 2.91)	0.37	68
Cohort study	10	0.46 (− 1.28 to 2.20)	< 0.01	74	16	0.09 (− 1.52 to 1.69)	0.92	78
Cross-sectional study	10	− 0.56 (− 4.21 to 3.10)	< 0.01	91	9	1.47 (− 1.56 to 4.50)	0.34	91
1,25(OH)_2_D_3_ among disease patients and healthy controls								
Maturity								
Adults (> 18 years old)	5	0.31 (− 12.88 to 13.50)	< 0.01	96	3	− 2.94 (− 7.25 to 1.38)	0.11	55
Children (< 18 years old)	3	8.64 (− 14.08 to 31.35)	< 0.01	99	2	16.54 (− 2.85 to 35.94)	0.01	84
Vitamin D assessment tool								
CPBA	1	15.70 (15.20 to 16.20)	NR	NR	1	− 0.80 (− 1.86 to 0.26)	NR	NR
HPLC	1	− 8.62 (− 21.62 to 4.38)	NR	NR	NR	NR	NR	NR
RIA	5	3.07 (− 13.33 to 19.47)	< 0.01	97	3	4.31 (− 20.38 to 28.99)	< 0.01	97
NR	1	3.20 (− 1.16 to 7.56)	NR	NR	1	5.30 (− 9.49 to 20.09)	NR	NR
Vitamin D supplementation								
No	8	3.47 (− 7.72 to 14.66)	< 0.01	98	5	3.76 (− 8.36 to 15.87)	< 0.01	96
Study design								
Case–control study	6	3.95 (− 9.09 to 16.98)	< 0.01	95	4	4.60 (− 15.56 to 24.77)	< 0.01	96
Cohort study	2	2.14 (− 24.51 to 28.80)	< 0.01	100	1	− 0.80 (− 1.86 to 0.26)	NR	NR
1,25(OH)_2_D_3_ among disease patients and non-healthy controls								
Maturity								
Adults (> 18 years old)	6	6.77 (− 2.30 to 15.84)	< 0.01	98	4	− 10.48 (− 21.86 to 0.89)	< 0.01	96
Children (< 18 years old)	3	1.40 (− 9.11 to 11.90)	0.06	64	3	− 1.40 (− 11.90 to 9.11)	0.06	64
Vitamin D assessment tool								
CPBA	2	6.07 (− 15.64 to 27.79)	< 0.01	94	2	− 6.07 (− 27.79 to 15.64)	< 0.01	94
HPLC + RIA	1	− 0.08 (− 4.59 to 4.43)	NR	NR	0	NR	NR	NR
RIA	4	0.87 (− 1.14 to 2.87)	0.11	55	3	− 3.51 (− 10.10 to 3.09)	0.11	55
NR	2	10.93 (− 13.44 to 35.31)	< 0.01	86	2	− 10.93 (− 35.31 to 13.44)	< 0.01	86
Vitamin D supplementation								
No	9	5.05 (− 2.42 to 12.52)	< 0.01	97	7	− 6.71 (− 15.30 to 1.88)	< 0.01	94
Study design								
Case–control study	6	0.60 (− 1.36 to 2.56)	0.26	23	5	− 1.00 (− 4.08 to 2.08)	0.17	37
Cohort study	2	16.57 (15.47 to 17.66)	0.25	24	2	− 16.57 (− 17.66 to − 15.47)	0.25	24
Cross-sectional study	1	− 0.08 (− 4.59 to 4.43)	NR	NR	0	NR	NR	NR

*CPBA* competitive protein binding assay, *RIA* radioimmunoassay, *ECLIA* electrochemiluminescence immunoassay, *ELISA* enzyme-linked immunosorbent assay, *CLIA* chemiluminescence, *HPLC* high performance liquid chromatograph, *LC–MS* liquid chromatograph mass spectrometer, *NR* not reported

**Fig. 3:**
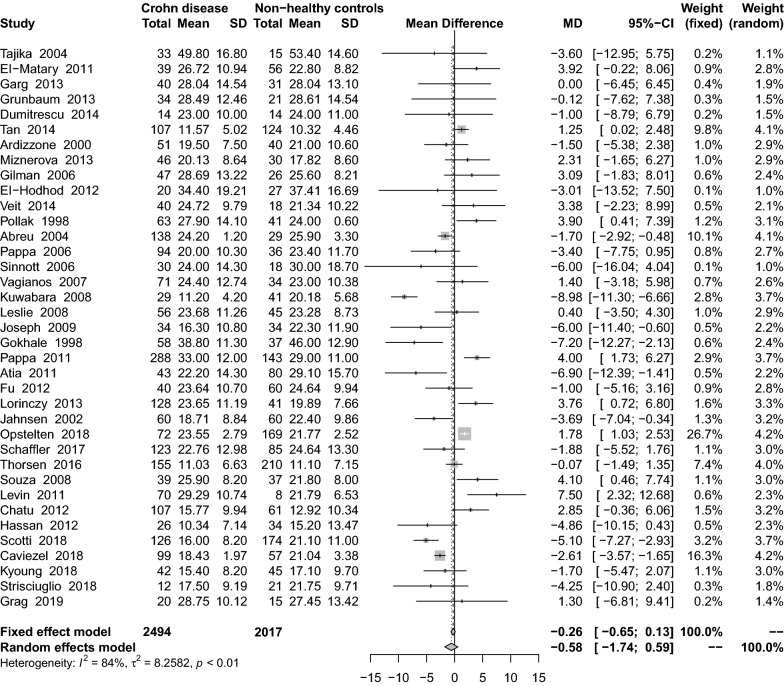
Mean difference of serum 25(OH)D levels among patients with Crohn’s disease compared with non-healthy controls

The discussion between CD and UC about serum 25(OH)D levels were identified in thirty-seven studies [16, 18, 27–30, 32, 34, 36–42, 44–48, 50–52, 54, 56, 58, 61, 62, 64, 66–68, 71, 73, 75–77], which included a total of 2494 CD patients and 2017 non-healthy controls. The analysis revealed no significant difference in average serum 25(OH)D levels between the two groups (MD: − 0.58 ng/mL; 95% CI − 1.74 to 0.59) (Fig. 3). There was significant heterogeneity among the studies (I^2^ = 84%, P < 0.01). Subgroup analysis showed that only ECLIA (MD: 1.34 ng/mL; 95% CI 0.17–2.52) and the use of VitD supplementation (MD: 2.36 ng/mL; 95% CI 1.46–3.25) were statistically significant (Table 2). In sensitivity results, the residual results were unchanged after excluding small sample studies (MD: − 0.51 ng/mL; 95% CI − 1.69 to 0.66) or excluding studies with lower quality score (MD: − 0.90 ng/mL; 95% CI − 2.12 to 0.31).

### Findings from the meta-analysis of 1,25(OH)_2_D_3_ levels in Crohn’s patients

**Fig. 4:**
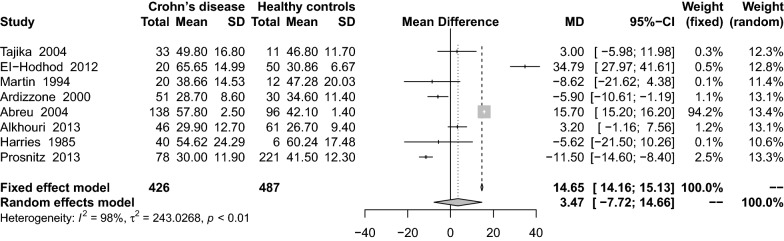
Mean difference of serum 1,25(OH)_2_D_3_ levels among patients with Crohn’s disease compared with healthy controls

Eight studies [26, 29, 32, 34, 46, 55, 59, 70] reported average serum 1,25(OH)_2_D_3_ concentrations in Crohn’s patients, and these were higher in the CD group in comparison with the healthy control group (MD: 3.47 pg/mL; 95% CI − 7.72 to 14.66) (Fig. 4). There was significant heterogeneity among the studies (I^2^ = 98%, P < 0.01). Subgroup analysis showed that the CPBA (MD: 15.70 ng/mL; 95% CI 15.20–16.20) was the only statistically significant variable (Table 2).

In sensitivity results, the residual results were unchanged after excluding small sample studies (MD: 5.02 ng/mL; 95% CI − 6.86 to 16.90) or excluding studies with lower quality score (MD: 3.46 ng/mL; 95% CI − 9.58 to 16.49).

**Fig. 5:**
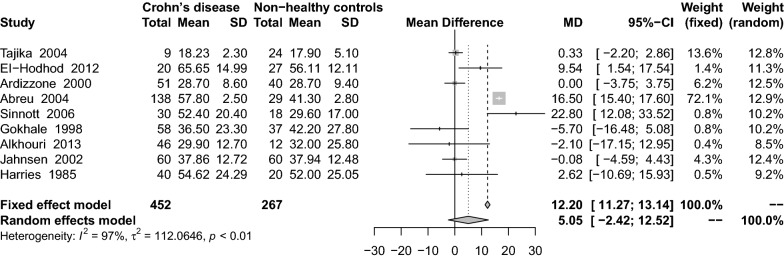
Mean difference of serum 1,25(OH)_2_D_3_ levels among patients with Crohn’s disease compared with non-healthy controls

In 9 included studies [26, 28–30, 32, 34, 38, 46, 59], the combined effect of the 1,25(OH)_2_D_3_ concentration on the comparison between CD patients and UC group was 5.05 pg/mL (95% CI − 2.42 to 12.52) (Fig. 5). There was significant heterogeneity among the studies (I^2^ = 97%, P < 0.01). Subgroup analysis showed that only the cohort study design (MD: 16.57 ng/mL; 95% CI 15.47–17.66) was statistically significant (Table 2). Sensitivity analysis results remained unchanged after the removing studies of lower quality score (MD: 3.56 ng/mL; 95% CI − 4.78 to 11.91).

### Findings from a meta-analysis of serum 25(OH)D levels in UC patients

**Fig. 6:**
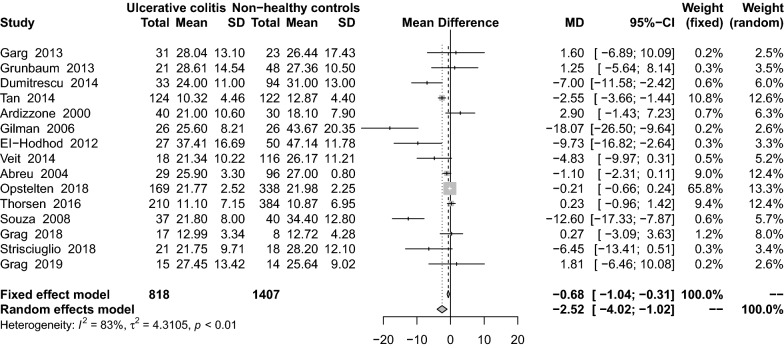
Mean difference of serum 25(OH)D levels among patients with ulcerative colitis compared with healthy controls

A meta-analysis of 15 studies [16, 29, 34, 36, 46, 54, 61, 62, 64, 66, 68, 71, 74, 76, 77] on serum 25(OH)D levels in both UC and healthy controls showed that patients with UC had lower levels of serum 25(OH)D than did the controls (MD: − 2.52 ng/mL; 95% CI − 4.02 to − 1.02) (Fig. 6). These studies had high heterogeneity (I^2^ = 83%, P < 0.01). Subgroup analysis showed that the following variables were statistically significant: adults (MD: − 2.38 ng/mL; 95% CI − 4.20 to − 0.56), HPLC (MD: − 7.00 ng/mL; 95% CI − 11.58 to − 2.42), lack of VitD supplementation (MD: − 3.29 ng/mL; 95% CI − 4.99 to − 1.60), and cross-sectional study design (MD: − 18.07 ng/mL; 95% CI − 26.50 to − 9.64) (Table 2). Sensitivity analysis results was stabilization after small sample studies were removed (MD: − 2.94 ng/mL; 95% CI − 4.55 to 1.33).

**Fig. 7:**
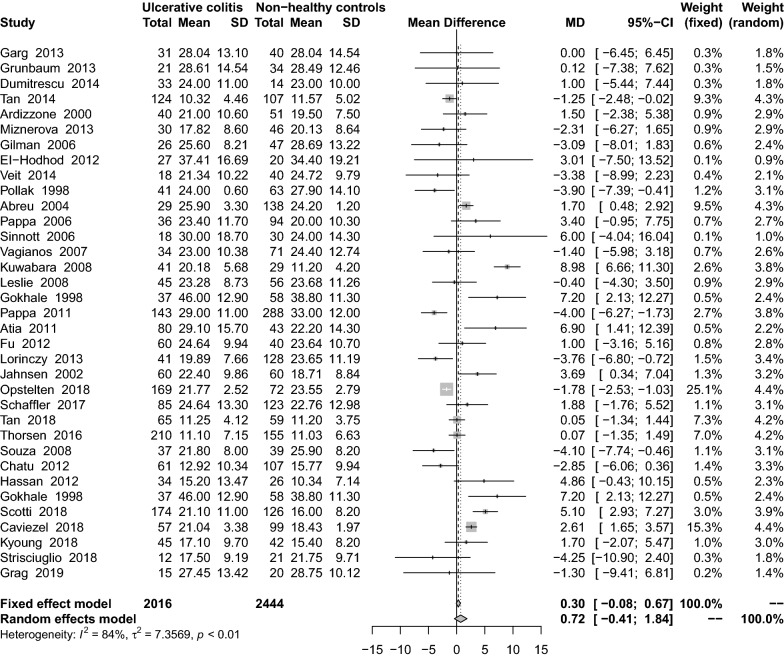
Mean difference of serum 25(OH)D levels among patients with ulcerative colitis compared with non-healthy controls

There was almost no difference between UC and CD in 34 studies [16, 18, 27, 29–31, 34, 36–41, 46–48, 50–52, 54, 56, 58, 61, 62, 64, 66–68, 71, 73, 75–77] investigating VitD levels (MD: 0.75 ng/mL; 95% CI − 0.44 to 1.94) (Fig. 7). These studies had high heterogeneity (I^2^ = 84%, P < 0.01). Subgroup analysis showed that ECLIA (MD: − 1.34 ng/mL; 95% CI − 2.52 to − 0.17), HPLC (MD: 3.69 ng/mL; 95% CI 0.34–7.04), lack of VitD supplementation (MD: − 2.11 ng/mL; 95% CI − 3.69 to − 0.53), and the use of VitD supplementation (MD: 0.71 ng/mL; 95% CI − 0.63 to 2.05) were statistically significant (Table 2). Sensitivity analysis results remained stable after the removal of small samples (MD: − 0.88 ng/mL; 95% CI − 0.34 to 2.10) or lower quality score (MD: 0.72 ng/mL; 95% CI − 0.52 to 1.96).

### Findings from the meta-analysis of 1,25(OH)_2_D_3_ levels in UC patients

**Fig. 8:**
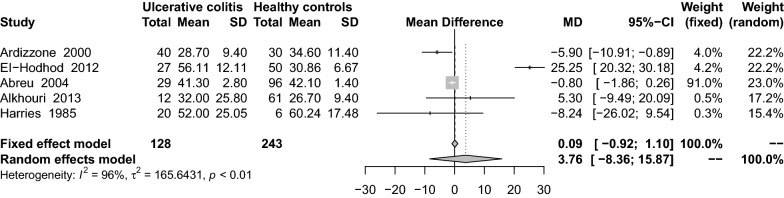
Mean difference of serum 1,25(OH)_2_D_3_ levels among patients with ulcerative colitis compared with healthy controls

Five studies [26, 29, 34, 46, 59] reporting on levels of 1,25(OH)_2_D_3_ in UC and healthy control groups found higher levels of 1,25(OH)_2_ D_3_ in the UC group than in the control group (MD: 3.76 pg/mL; 95% CI − 8.36 to 15.57) (Fig. 8). There was significant heterogeneity among the studies (I^2^ = 96%, P < 0.01). None of the results of the subgroup analyses from these studies were statistically significant (Table 2). Sensitivity analysis results remained unchanged after small samples were removed (MD: 3.40 ng/mL; 95% CI − 10.26 to 17.06).

**Fig. 9:**
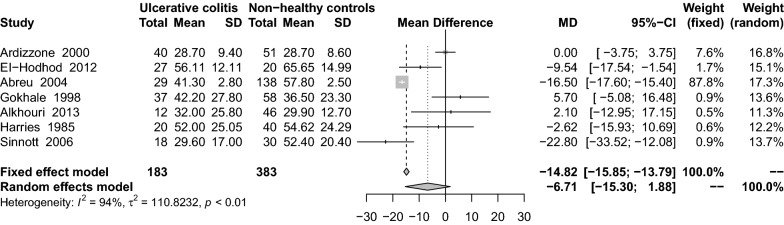
Mean difference of serum 1,25(OH)_2_D_3_ levels among patients with ulcerative colitis compared with non-healthy controls

Overall, when all seven eligible studies [26, 29, 30, 34, 38, 46, 59] were analyzed using a random-effects model, the results showed that VitD levels were lower in patients with UC than in CD (MD: − 6.71 pg/mL; 95% CI − 15.30 to 1.88) (Fig. 9). There was significant heterogeneity among the studies (I^2^ = 94%, P < 0.01). Subgroup analysis showed that only the cohort studies (MD: − 16.57 ng/mL; 95% CI − 17.66 to − 15.47) were statistically significant (Table 2). Sensitivity analysis results remained unchanged after small samples were removed (MD: − 5.09 ng/mL; 95% CI − 15.28 to 5.10).

### Publication bias

For the meta-analyses, publication bias was not assumed, as all funnel plots were essentially symmetrical.

## Discussion

There are several competing views on the link between VitD deficiency and IBD in the literature. For UC, Ulitsky et al. [17] reported that VitD deficiency is not associated with UC, but another study [78] reported a correlation. With regard to CD, Khalili et al. [79] reported that VitD deficiency was associated with CD, but the Grunbaum’s [16] study did not. To explore this controversy, we performed a pooled meta-analysis to determine the status of VitD in the serum of healthy and non-healthy controls.

Vitamin D is the only fat-soluble vitamin that may provide potential effects in treating IBD [7]. From our meta-analysis, we have concluded that VitD levels are strongly associated with IBD. Our meta-analysis found that patients with CD and UC had mean lower levels of 25(OH)D than did healthy populations; however, there was no significant difference in serum 25(OH)D levels between CD and UC patients. So VitD levels may be independent of disease type. This can be explained by insufficient intake, insufficient absorption or excessive loss of VitD in patients with IBD [13]. When comparing the mean levels of 1,25(OH)_2_D_3_, we found that patients with CD and UC did not lack 1,25(OH)_2_D_3_, and, in fact, patients with CD and UC had higher levels of VitD than healthy populations. Moreover, the average concentration of 1,25(OH)_2_D_3_ in CD patients was significantly higher than in patients with UC.

Current studies [80–82] have suggested that VitD plays a role in IBD-specific complications. The best indicator of VitD status is serum 25(OH)D because it closely reflects both dietary intake and the amount of sunlight exposure [83], and 25(OH)D has a half-life of 12 to 19 days [5, 13], however, 1,25(OH)_2_D_3_ has a short half-life of 4 to 20 h and is not a reliable indicator of the total amount of vitamin D in the body [84]. Although the serum 1,25(OH)_2_D_3_ content of IBD patients was higher than that of healthy populations, we cannot ignore the importance of 1,25(OH)_2_D_3_. In accordance with our findings, Abreu’s study [34] also demonstrated that IBD patients have high levels of 1,25(OH)_2_D_3_, especially in CD patients. It has been suggested that elevated 1,25(OH)_2_D_3_ may be a direct cause of bone loss or act as a surrogate marker for the type of intestinal inflammation that results in osteoporosis. In addition, in the presence of intestinal inflammation, an increase in the number of lamina propria monocytes, combined with the availability of 25(OH)D as a 1a-hydroxylase substrate, resulted in increased levels of 1,25(OH)_2_D_3_ [34, 85]. In our study, we also found that the level of 1,25(OH)_2_D_3_ in patients with CD was significantly higher than that in patients with UC. However, in some studies, we also found that the serum level of 1,25(OH)_2_D_3_ was lower in IBD patients than in healthy control groups. This may be due to improved BMD after remission of IBD, making 1,25(OH)_2_D_3_ normal.

Based on the subgroup analysis of age, VitD deficiency was more common in adults and children with IBD. Although, there was no significant difference in VitD levels between adults and children, whether they were in an IBD or a healthy control group. In children, El-Matary et al. [44] found that VitD levels were lower (though not statistically significant) in UC patients than in a CD group. However, in Veit’s study, 25(OH)D was significantly higher in children with CD than in children with UC [65]. In our subgroup analysis, we found no significant differences in vitamin D levels between CD and UC pediatric patients; and, we found the same results in adults. An association between IBD risk and pre-diagnosis predicted VitD status has been established in adult populations. There may be differences in genetic susceptibility and immunopathogenic pathways between childhood and adult onset IBD, because children with IBD seem to be a unique group with special characteristics that require highly skilled and specialized methods for diagnosis and treatment [76, 86, 87].

With VitD intake and foods meeting only 20% of total daily needs, it is important to educate people about the importance of introducing foods rich in vitamin D into their daily diet [88]. The RDA is 400 international units (IU) or 10 ng for male and female infants (i.e., less than 1 year old), 600 IU or 15 ng for all male and female individuals from 1 to 70 years old, and 800 IU or 20 ng for those over 70 years old [89]. Dietary supplements are generally considered to be a rapid form of VitD supplementation, and the total intake of VitD always reflects the combined contribution of the food source and the supplement to the diet. VitD can be found in VitD_2_ or VitD_3_; however, the former is rarely used as a fortifier in dietary supplements [90, 91]. Increasing VitD in foods may be the best way to increase intake, but it does not significantly increase serum 25(OH)D levels. We believe that VitD supplements should be used to increase serum VitD levels more quickly and directly. Of course, dietary supplements with high VitD content may help improve the low VitD levels in patients with IBD.

VitD supplementation has been shown to reduce the recurrence of some immune-mediated diseases [92, 93], and adverse events associated with VitD supplementation is relatively low. VitD supplementation reduced clinical recurrence from 29 to 13% (P = 0.06) [94]. We measured VitD supplementation in the analysis, which was found in 12 studies. Jorgensen [57] found that CD patients reported taking VitD supplements in winter, and their levels of 25(OH)D were significantly higher than non-users. This further confirms the views of Pappa [47] and Grunbaum [16] who suggested that higher doses may yield better results. Other studies have shown that VitD is more necessary in winter and that large amounts of it are more effective (even up to 10,000 IU/day) [95–97]. High doses of VitD_3_ supplements (10,000 IU/day) may significantly reduce clinical recurrence and significantly improve quality of life [94, 98–100]. VitD_3_ is formed by exposure of the skin to sunlight [101]. In winter, when sunlight is scarce, VitD should be taken. Notably, in several studies more IBD patients were found to be taking VitD supplements, and subsequently tended to have higher total daily oral intake of vitamin D [43, 54, 77]. Since there is not enough trial data investigating different doses of vitamin D supplements, large, well-designed randomized controlled trials using different doses of vitamin D supplements are needed to help better understand the therapeutic significance of vitamin D in IBD.

In addition, we found that different VitD measurement tools may affect the final results. After our analysis, VitD deficiency in IBD patients measured by ELISA and HPLC was found to be more severe (though not statistically significant) in comparison to control groups. Therefore, different VitD measurements may affect the results. There are different methods for the determination of 25(OH)D, including competitive binding protein assays, immunoassays (such as chemiluminescence immunoassays [CLIA]), high performance liquid chromatography (HPLC), and liquid chromatography-tandem mass spectrometry (LC-MS/MS) that are currently considered more accurate and accurate [102, 103]. A studies have shown that different methods of vitamin D measurement can affect the results of vitamin D measurement [104–107]. Therefore, I believe that the standardization of vitamin D measurement is helpful for the diagnosis and treatment of IBD. In addition, free 25(OH)D may reflect the status of biologically active vitamin D better than total 25(OH)D [108]. Recent studies have shown that patients with IBD have normal or even higher levels of free 25(OH)D, despite a total deficiency of 25(OH)D [76]. Measuring free 25(OH)D may establish a relationship between IBD and vitamin D.

In terms of study design, a significant difference was found in the cohort studies for 1,25(OH)_2_D_3_ between the diseased patients and non-healthy controls, but this result may have been caused by small sample sizes. There was no significant difference between study designs among the other groups. Therefore, different research designs did not affect the final results.

It is unclear whether VitD deficiency is a consequence of IBD or a contributing factor to its pathogenesis. However, VitD may be an important mediator in the pathogenesis of CD and possibly UC [109]. Though our research found a relationship between the VitD deficiency and IBD, the relationship with UC was not obvious in some respects. It is possible that VitD deficiency is more closely related to celiac disease, and that the disease activity of celiac disease promotes the process of UC.

One advantage of this meta-analysis was that it included a large number of subjects, including CD and UC subjects, which examined the associations between 25(OH)D and 1,25(OH)_2_D_3_ levels, and considered healthy and non-healthy controls in their analyses. Furthermore, it was possible to perform subgroup analyses according to age group, VitD assessment tools, VitD supplementation and study design. In our sensitivity analysis, we excluded small samples and low-scoring studies to see if the results were altered. However, this meta-analysis has some limitations. First, there was no subgroup analysis based on gender, season, race, or disease activity, as there was not enough data. Second, although funnel plots showed no significant publication bias, there may still be publication biases in the retrieved articles. Third, there was no unified diagnostic standard for IBD in the included studies, which may have greatly increased the false positive rate and affected the results of the included studies. Fourth, the relevant parties of RDA cannot do in-depth analysis due to various objective reasons.

## Conclusions

In summary, we found that VitD levels were inversely related to CD and UC. Serum levels of 25(OH)D_3_ were lower in these patients than in healthy controls, and more than half of the patients had insufficient vitamin D levels; however, the serum level of 1,25(OH)_2_ D_3_ was higher than that of healthy controls. Our analysis indicates that attention should be paid to VitD levels to prevent the occurrence of IBD. In clinical practice, IBD patients should supplement their diets with VitD and be aware of the effects different seasons have on VitD content. In follow-up studies, vitamin D may be used as a treatment for IBD, or as an adjunctive therapy. We believe our research can provide a reference point for other scholars; however, our results cannot clarify the pathogenesis or suggest a cure for IBD. Rather, these results should provide directions for future research, as more exploration is needed.

## Supplementary information

**Additional file 1: Method S1**. Search strategy.

## Data Availability

Not applicable.
