# Supplementary material for: Vitamin D deficiency associated with Crohn’s disease and ulcerative colitis: a meta-analysis of 55 observational studies
Source: J Transl Med. 2019 Sep 23;17:323. doi: 10.1186/s12967-019-2070-5 (PMC6757415; doi:10.1186/s12967-019-2070-5)
Supplement: Supplementary file 1 — Additional file 1: Method S1. Search strategy. [file 12967_2019_2070_MOESM1_ESM.docx]

**Additional file 1**. Search Strategy

**1. PubMed, April 8, 2019**

("vitamin d"[MeSH Terms] OR "vitamin d"[All Fields] OR "ergocalciferols"[MeSH Terms] OR "ergocalciferols"[All Fields]) OR ("25-hydroxyvitamin D"[Supplementary Concept] OR "25-hydroxyvitamin D"[All Fields] OR "25 hydroxyvitamin d"[All Fields] OR "calcifediol"[MeSH Terms] OR "calcifediol"[All Fields]) OR (25[All Fields] AND ("hydroxide ion"[Supplementary Concept] OR "hydroxide ion"[All Fields] OR "oh"[All Fields]) AND ("vitamins"[Pharmacological Action] OR "vitamins"[MeSH Terms] OR "vitamins"[All Fields] OR "vitamin"[All Fields]))) AND (("colitis, ulcerative"[MeSH Terms] OR ("colitis"[All Fields] AND "ulcerative"[All Fields]) OR "ulcerative colitis"[All Fields] OR ("ulcerative"[All Fields] AND "colitis"[All Fields])) OR ("crohn disease"[MeSH Terms] OR ("crohn"[All Fields] AND "disease"[All Fields]) OR "crohn disease"[All Fields] OR ("crohn's"[All Fields] AND "disease"[All Fields]) OR "crohn's disease"[All Fields]) OR ("inflammatory bowel diseases"[MeSH Terms] OR ("inflammatory"[All Fields] AND "bowel"[All Fields] AND "diseases"[All Fields]) OR "inflammatory bowel diseases"[All Fields] OR ("inflammatory"[All Fields] AND "bowel"[All Fields] AND "disease"[All Fields]) OR "inflammatory bowel disease"[All Fields])) / (864)

**2. EMbase, April 8, 2019**

'ergocalciferol'/exp OR ergocalciferol AND [1966-2015]/py OR '25 hydroxycholecalciferol' OR 'sunshine vitamin' OR '25 hydroxyvitamin d' OR 'vitamin d'/exp OR 'vitamin d' OR 'calcifediol' AND ('inflammatory bowel diseases'/exp OR 'inflammatory bowel diseases' OR 'crohns disease'/exp OR 'crohns disease' OR 'ulcerative colitis'/exp OR 'ulcerative colitis') AND ('case control study' OR 'cohort analysis' OR 'retrospective study' OR 'observational study') / (273)

**3. EBSCO*host* CINAHL, April 8, 2019**

1. ergocalciferol /(169)
2. vitamin d /(11441)
3. MH vitamin d/(8091)
4. 25-hydroxyvitamin d /(1508)
5. Calcifediol /(6)
6. S1 OR S2 OR S3 OR S4 OR S5 /(11532)
7. inflammatory bowel disease /(3805)
8. crohns disease /(3440)
9. crohn's disease /(2125)
10. ulcerative colitis /(2436)
11. S7 OR S8 OR S9 OR S10 /(7213)
12. S6 AND S11 / (248)
